# Distinct mucosal and systemic immunological characteristics in transgender women potentially relating to HIV acquisition

**DOI:** 10.1172/jci.insight.169272

**Published:** 2023-08-22

**Authors:** Alexandra Schuetz, Michael J. Corley, Carlo Sacdalan, Yuwadee Phuang-Ngern, Thitiyanun Nakpor, Tanyaporn Wansom, Philip K. Ehrenberg, Somchai Sriplienchan, Rasmi Thomas, Nisakorn Ratnaratorn, Suchada Sukhumvittaya, Nipattra Tragonlugsana, Bonnie M. Slike, Siriwat Akapirat, Suteeraporn Pinyakorn, Rungsun Rerknimitr, Alina P.S. Pang, Eugène Kroon, Nipat Teeratakulpisan, Shelly J. Krebs, Nittaya Phanuphak, Lishomwa C. Ndhlovu, Sandhya Vasan, on behalf of the RV304/SEARCH013 Study Team

**Affiliations:** 1 Armed Forces Research Institute of Medical Sciences, Bangkok, Thailand.; 2 US Military HIV Research Program, Walter Reed Army Institute of Research, Silver Spring, Maryland, USA.; 3 Henry M. Jackson Foundation for the Advancement of Military Medicine Inc., Bethesda, Maryland, USA.; 4 Division of Infectious Diseases, Department of Medicine, Weill Cornell Medicine, New York, New York, USA.; 5 SEARCH Research Foundation, Bangkok, Thailand.; 6 Sisters Foundation, Pattaya, Thailand.; 7 Department of Medicine, Faculty of Medicine, Chulalongkorn University, Bangkok, Thailand.; 8 Institute of HIV Research and Innovation, Bangkok, Thailand.; 9 The RV304/SEARCH013 Study Team is detailed in Supplemental Acknowledgments.

**Keywords:** AIDS/HIV, Immunology, Cellular immune response, Sex hormones, T cells

## Abstract

Transgender women (TGW) are disproportionally affected by HIV infection, with a global estimated prevalence of 19.9%, often attributed to behavioral risk factors, with less known about biological factors. We evaluated potential biological risk factors for HIV acquisition in TGW at the sites of viral entry by assessing immune parameters of the neovaginal surface and gut mucosa. The neovagina in TGW, compared with the vagina in cisgender women (CW), shows distinct cell composition and may pose a more inflammatory environment, evidenced by increased CD4^+^ T cell activation and higher levels of soluble markers of inflammation (C-reactive protein, soluble CD30). Increased inflammation may be driven by microbiome composition, as shown by a greater abundance of Prevotella and a higher Shannon Diversity Index. In addition, we have observed higher frequency of CD4^+^CCR5^+^ target cells and decreased DNA methylation of the *CCR5* gene in the gut mucosa of TGW compared with CW and men who have sex with men, which was inversely correlated with testosterone levels. The rectal microbiome composition in TGW appears to favor a proinflammatory milieu as well as mucosal barrier disruption. Thus, it is possible that increased inflammation and higher frequencies of CCR5-expressing target cells at sites of mucosal viral entry may contribute to increased risk of HIV acquisition in TGW, with further validation in larger studies warranted.

## Introduction

HIV has differential impacts on communities and key populations. In a large meta-analysis, the odds ratio for being infected with HIV in transgender women (TGW) compared with all adults of reproductive age was 48.8 (95% CI 21.2–76.3) irrespective of country income level, with a global pooled estimated prevalence of 19.9% (1, 2). This statistic may be driven by several factors, including high rates of condom-less anal intercourse and sexually transmitted infections (STIs) and poorer access to HIV testing and preventive health care services due to increased stigma and legal discrimination. Risk is further increased by psychosocial health problems including mental health issues, substance use, and targeted violence (3, 4).

However, there is little research addressing whether this may also be compounded by increased biological risk for HIV acquisition due to biological factors unique to TGW as part of the male-to-female transition. To minimize secondary sex characteristics generally associated with men, TGW undergo feminizing hormone treatment to increase female characteristics, which includes the usage of progestogens, estrogens, and antiandrogens (5). Recommended feminizing hormone regimens are composed of estrogen to promote female secondary sexual characteristics and androgen-lowering drugs to inhibit male secondary sexual characteristics by decreasing endogenous testosterone production or testosterone activity (6, 7). However, in Thailand, there is high use of feminizing hormone treatment reported outside the reference regimen as these drugs can be purchased without a medical prescription (8). Progesterone and estrogen are known to have differential effects on mucosal epithelial barriers, modulating cellular expression of CCR5, the primary co-receptor for entry of R5 tropic HIV into CD4^+^ T cells (9), and α4β7, a cellular gut-homing marker (10), as well as on vaginal epithelial thickness (11, 12). Sex hormone levels are also linked to inflammation, induced by the impact they have on the composition of microbial communities (13). In addition, multiple studies have demonstrated that the microbiome is a major determinant of the local immune environment, with sex hormones such as progesterone estradiol and testosterone directly impacting the microbiome composition in the gut (14, 15). In general, cisgender men (CM) exhibit a lower microbial diversity than cisgender women (CW), with healthy CW showing predominantly Firmicutes and CM a higher abundance of Bacteroides Prevotella (16, 17). Sex hormones directly regulate bacterial growth; e.g., *Prevotella* *intermedius* takes up estradiol and progesterone, favoring bacterial expansion and in turn affecting inflammation (18). Furthermore, the intestinal mucosa is known to be steroidogenic, and colonic epithelial cells express estrogen receptor β, making the intestinal mucosa likely to be affected by feminizing hormone treatment (19, 20).

To further affirm gender identity, TGW may undergo a variety of surgical procedures such as use of injectable fillers, breast augmentation, and vaginoplasty (7). Those without affordable access to sterile liquid silicone may use substances such as oil or petroleum jelly as injectable fillers (7), which may predispose to systemic immune activation and render CD4^+^ T cells more susceptible to HIV infection. Gender affirmation surgery (GAS) by vaginoplasty aims to create a functional vagina which can be achieved by a variety of surgical techniques including penile skin inversion, colonic flap, or peritoneal flap vaginoplasty (21, 22). Currently, penile skin inversion is the most widely used technique for GAS. The cell composition of the skin creates a nonlubricated neovagina due to lack of columnar epithelial cells that is prone to stenosis and requires regular dilatation and intensive longitudinal postsurgical care (7, 23). It is therefore possible that this creates a more inflammatory milieu both due to mechanical abrasion and potentially substandard long-term care. In addition, neovaginas reconstructed by penile skin inversion have diverse polymicrobial communities that can increase immune activation and decrease epithelial barrier function (24). Taken together, it is plausible that these changes may predispose to a more favorable neovaginal environment for HIV transmission, given that cervicovaginal inflammation in CW predisposes to increased HIV acquisition (25, 26); however, it is of note that HIV entry in the female genital tract (FGT) occurs mainly at the transition zone/cervix (27).

More detailed and systematic studies are therefore needed to better understand potential biological factors unique to TGW that could influence the risk of HIV acquisition. Here, we report evidence that the unique biology of TGW sets them apart from CW and men who have sex with men (MSM). This includes (i) a higher inflammatory environment in the neovagina reconstructed by penile skin inversion that may be driven by a highly diverse microbiome, (ii) an increase in CCR5 expression in the intestinal mucosa related to hormonal levels, and (iii) distinct rectal microbiome composition impacting gut mucosal homeostasis.

## Results

### Study participants.

We compared immunological parameters of mucosal surfaces that could contribute to the increased risk of HIV acquisition reported in TGW than CW and MSM. In the RV304/SEARCH013 cohort in Thailand, participants living without HIV were enrolled cross-sectionally, including 10 TGW, 10 CW who were not taking hormonal contraceptives, and 10 MSM (Table 1). Median age of TGW was 31 years (range 25, 49 years), 37 years for CW (range 26, 42 years) and 27 years (range 19, 39 years) for MSM (Kruskal-Wallis *P* = 0.02). Mean lifetime sexual partners were highest among TGW with a median of 35 (range 1, 4,500) compared with CW (median 3; range 1, 11) and MSM (median 25; range 3, 70; Kruskal-Wallis *P* = 0.002). All 10 TGW were after GAS, with a median time since GAS of 7.4 years (range 2, 20 years), and all vaginoplasty procedures to create a functional neovagina were performed by penile skin inversion. Among TGW, median age of initial gender dysphoria was 5.5 years, expression of feminine gender through external appearance 14.5 years, hormone initiation 15 years, and GAS 21.5 years (Supplemental Figure 1A; supplemental material available online with this article; https://doi.org/10.1172/jci.insight.169272DS1). As inclusion criteria, all TGW were on continuous feminizing hormone treatment for a minimum of 3 months (median: 6.6 years; range 0.5, 16 years). All 10 TGW reported the usage of oral estrogen (median: 2 mg; range 0.25, 10 mg) for feminizing hormone treatment and 6/10 concomitant intramuscular progesterone usage (median: 217 mg; range 150, 250 mg). None of the 10 CW were taking hormonal contraceptives at the time of the study. Four of 10 TGW and 4/10 MSM reported a history of sex work, with a history of sexually transmitted diseases in 2/10 TGW and 3/10 MSM. In contrast, CW did not report any previous history of sex work and sexually transmitted diseases. While MSM and CW exclusively reported receptive anal and vaginal intercourse, respectively, 7/10 TGW reported to engage in receptive anal and neovaginal intercourse, with 2/10 reporting only neovaginal and 1/10 only receptive anal intercourse (Supplemental Figure 1B).

Median CD4^+^ count was 1,179 cells/mm^3^ (range 594, 2,059), 826 cells/mm^3^ (range 39, 1,196), and 900 cells/mm^3^ (range 499, 1,261) in TGW, CW, and MSM, respectively (*P* = NS, Table 1). Median testosterone levels were 0.16 ng/mL (range 0.14, 0.27), 0.25 ng/mL (range 0.12, 0.43), and 4.02 ng/mL (range 1.15, 7.36; Kruskal-Wallis *P* < 0.001), and median estradiol E2 levels were 10.5 pg/mL (range 10, 94.6), 73.2 pg/mL (range 28.6, 385.9), and 15.2 pg/mL (range 10, 38.1; Kruskal-Wallis *P* < 0.001) in TGW, CW, and MSM, respectively (Table 1 and Supplemental Figure 1, C and D).

### Differential composition of CD4^+^ and CD8^+^ T cell populations in vaginal mucosa compared with neovaginal surfaces grafted by penile skin inversion.

**Figure 1:**
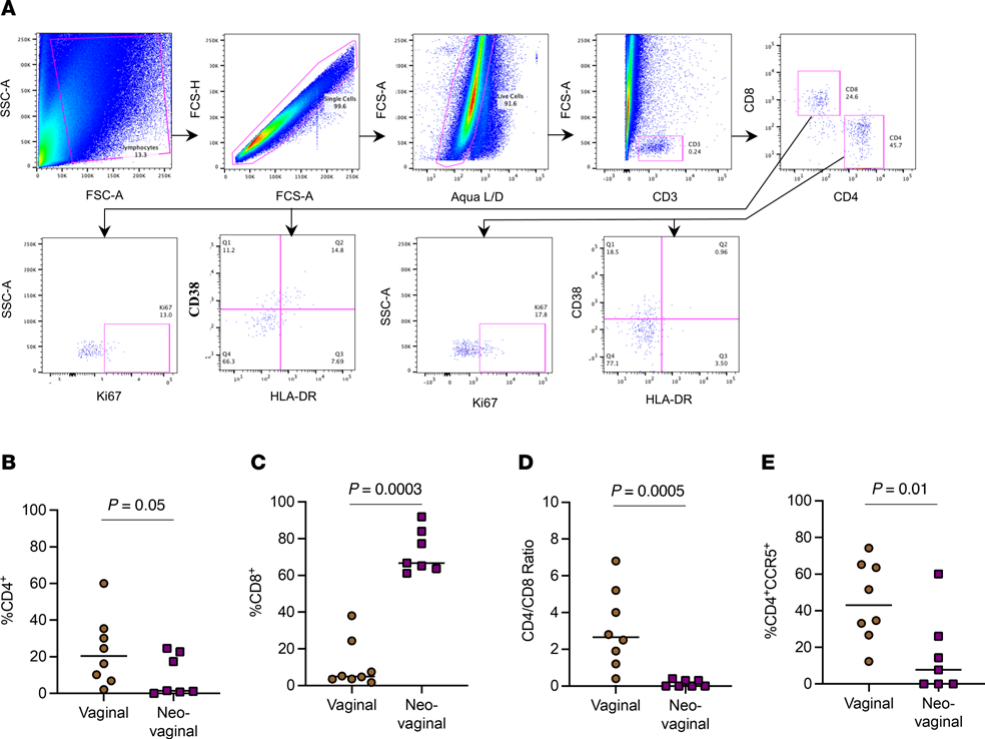
Frequency of CD4^+^ and CD8^+^ T cells differs between the vagina and the neovagina. (**A**) Example of parent gating strategy of freshly isolated neovaginal mononuclear cells. The staining profile of vaginal mononuclear cells from a representative volunteer is shown, indicating the frequency of activated CD4^+^ and CD8^+^ T cells by the expression of Ki67 and coexpression of CD38 and HLA-DR. (**B**) Frequency of vaginal (*n* = 8) and neovaginal (*n* = 7) CD4^+^ T cells and (**C**) CD8^+^ T cells, (**D**) CD4/CD8 T cell ratio, and (**E**) frequency of CD4^+^CCR5^+^ T cells are shown depending on sample availability. Difference between groups were analyzed using unpaired *t* test.

Establishing the immune profile of CD4^+^ T cells, the primary target cells of HIV in the FGT, is critical to better understand the risk of HIV acquisition (28). Several studies focusing on characterizing the CD4^+^ T cell subsets in the vaginal mucosa have shown that vaginal CD4^+^ T cells express high levels of CCR5 and are rapidly depleted in HIV/SIV infection (29). However, there is little known about the cellular composition of the surfaces of neovaginas in TGW created through different vaginoplasties (21, 24). To evaluate cell composition of the neovagina, grafted by penile skin inversion, we collected 2 sequential neovaginal and vaginal swabs, respectively, to identify and characterize different T cell subsets (Figure 1A). In the vaginal mucosa we observed a high frequency of CD4^+^ T cells (median 20.6%), and while the neovaginal surfaces also contained CD4^+^ T cells, they were observed in far lower frequencies (median 1.5%, *P* = 0.05; Figure 1B). Similarly, the vaginal mucosal contained a higher frequency of CD4^+^ T cells expressing the CCR5 HIV co-receptor compared with neovaginal surfaces (median: vaginal 43.1% vs. neovaginal 8.0%, *P* = 0.01; Figure 1E). Interestingly, we also observed a significantly lower frequency of CD4^+^CCR5^+^ T cells in the peripheral blood of TGW (median 8.4%) compared with CW (median 18.6%, *P* = 0.003), while there were no differences in the frequency of CD4^+^ and CD8^+^ T cells and the CD4/CD8 ratio (Supplemental Figure 2, A, D, and H). Surprisingly, CD8^+^ T cells were highly enriched on neovaginal surfaces (median 66.7%) compared with vaginal mucosa (median 3.6%; *P* = 0.0003), resulting in a significantly decreased CD4/CD8 ratio (median: vaginal 2.5 vs. neovaginal 0.01; *P* = 0.0005; Figure 1, C and D).

### Cellular and soluble inflammatory markers are distinct between the vaginal mucosa and the neovaginal surfaces.

**Figure 2:**
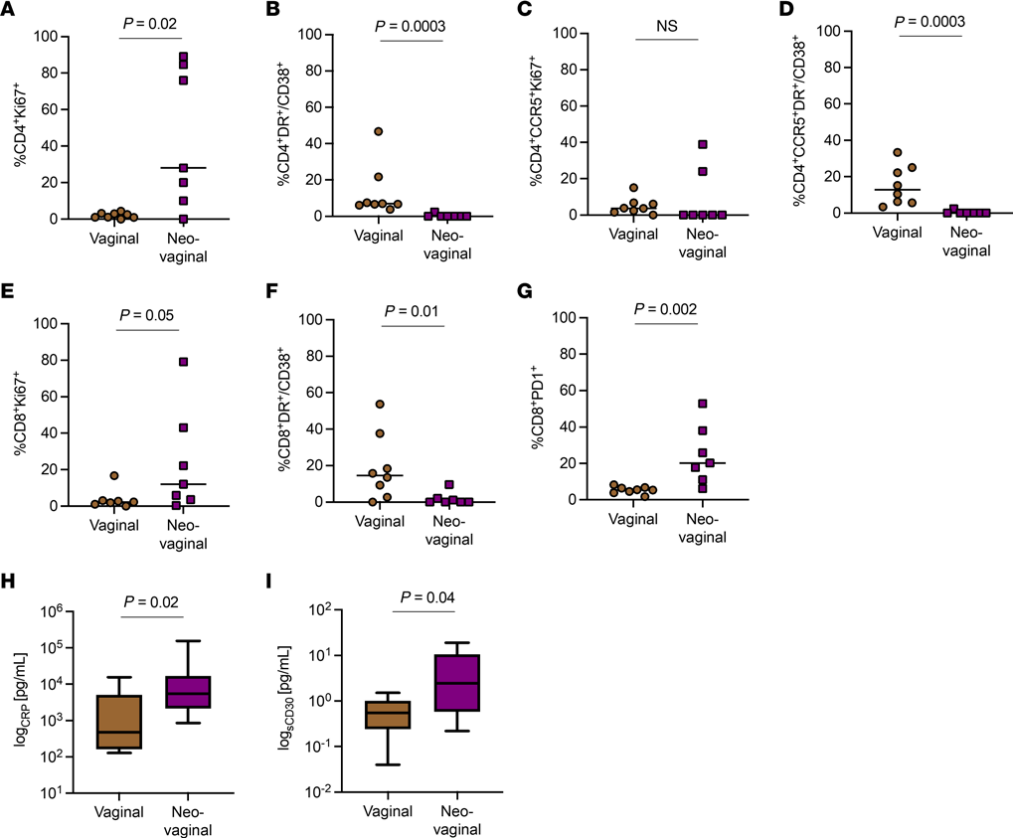
Cellular and soluble inflammation profiles differ between the vagina and the neovagina. Frequencies of cycling CD4^+^ (**A**) and CD8^+^ (**E**) T cells are increased in the neovagina (*n* = 7) compared with the vagina (*n* = 8), while there is no difference in the frequency of cycling CD4^+^CCR5^+^ (**C**) T cells observed between the vagina and the neovagina. In contrast, CD4^+^ (**B**), CD4^+^CCR5^+^ (**D**), and CD8^+^ (**F**) T cells in the vagina had a higher activation status compared with the neovagina indicated by the coexpression of HLA-DR and CD38. However, there was also a significant increase in PD-1–expressing CD8^+^ T cells observed in the neovagina (**G**), which was not seen in CD4^+^ T cells (*data not shown*). The only soluble biomarkers out of 18 tested that were significantly different between the vagina and the neovagina were the inflammation makers CRP (median: neovaginal 548 pg/mL vs. vaginal 480 pg/mL) (**H**) and sCD30 (median: neovaginal 2.45 pg/mL vs. vaginal 0.55 pg/mL) (**I**) that were significantly increased in neovaginal secretions (**H**). Differences between groups were analyzed using unpaired *t* tests.

Similar to the importance of the CD4^+^ T cell immune profiles in the vaginal mucosa, several studies also have shown that cellular and soluble markers of inflammation in the vagina significantly contribute to the risk of HIV acquisition (25, 30, 31). However, those data are scarce in relation to the inflammatory profile of the neovagina, with only a few studies attempting to assess this in TGW after GAS (24). Consequently, we quantified levels of T cell activation by measuring the frequency of HLA-DR/CD38 coexpression and the frequency of cycling T cells by the expression of Ki67, in the vaginal mucosa and neovaginal surfaces, respectively, in CD4^+^, CD4^+^CCR5^+^, and CD8^+^ T cells (Figure 1A). In CD4^+^ T cells the frequency of cycling cells was significantly increased in neovaginal surfaces compared with vaginal mucosa (median: neovaginal 28.0% vs. vaginal 1.8%; *P* = 0.02, Figure 2A). In contrast, CD4^+^ T cell activation, indicated by the coexpression of HLA-DR/CD38, was higher on vaginal compared with neovaginal CD4^+^ T cells (median: neovaginal 0.1% vs. vaginal 6.9%; *P* = 0.0003; Figure 2B). A similar trend was observed for CD4^+^CCR5^+^ T cells, where a significantly higher activation status was observed in vaginal CD4^+^CCR5^+^ T cells (median: neovaginal 0.1% vs. vaginal 12.8%; *P* = 0.0003; Figure 2D); however, there was no difference in the frequency of cycling CD4^+^CCR5^+^ T cells (median: neovaginal 0.1% vs. vaginal 3.7%; *P* = NS; Figure 2C). A higher frequency of Ki67-expressing and programmed cell death 1–expressing (PD-1–expressing) neovaginal CD8^+^ T cells was observed compared with those found in vaginal mucosa (median Ki67: neovaginal 12.0% vs. vaginal 2.3%; *P* = 0.05; Figure 2E; median PD-1: neovaginal 20.2% vs. vaginal 5.5%; *P* = 0.002; Figure 2G). The frequency of activated CD8^+^ T cells was also higher in vaginal mucosa compared with neovaginal surfaces (median: neovaginal 0.1% vs. vaginal 14.7%; *P* = 0.01; Figure 2F). There was no difference observed in the frequency of activated and cycling CD4^+^, CD4^+^CCR5^+^, and CD8^+^ T cells in peripheral blood in TGW and CW (Supplemental Figure 2).

Next, we explored the different levels of soluble inflammatory cytokines in vaginal and neovaginal secretions as elevated levels of inflammatory cytokines such as macrophage inflammatory protein 1β (MIP-1β), IP-10, and IL-8 have been previously linked to a higher risk of HIV acquisition in women (25). We observed that the levels of C-reactive protein (CRP) and soluble CD30 (sCD30) were significantly elevated in neovaginal secretions compared with vaginal secretions (median CRP: neovaginal 548 pg/mL vs. vaginal 480 pg/mL; *P* = 0.02; Figure 2H; median sCD30: neovaginal 2.45 pg/mL vs. vaginal 0.55 pg/mL; *P* = 0.04; Figure 2I). Other inflammatory cytokines tested that have previously been linked to a higher risk of HIV acquisition, including monocyte chemoattractant protein-1 (MCP-1), IP-10, MIP-1β, IL-8, TNFα-RI, and IFN-α2, were not different between vaginal and neovagina secretions (data not shown). In plasma there were no differences between biomarker levels in CW and TGW, except for MCP-1 plasma levels with a median of 0.26 pg/mL in TGW showing increased levels over a median 0.18 pg/mL in CW (*P* = 0.05; data not shown).

### Distinct composition of vaginal and neovaginal microbial communities relate to local inflammatory profiles.

**Figure 3:**
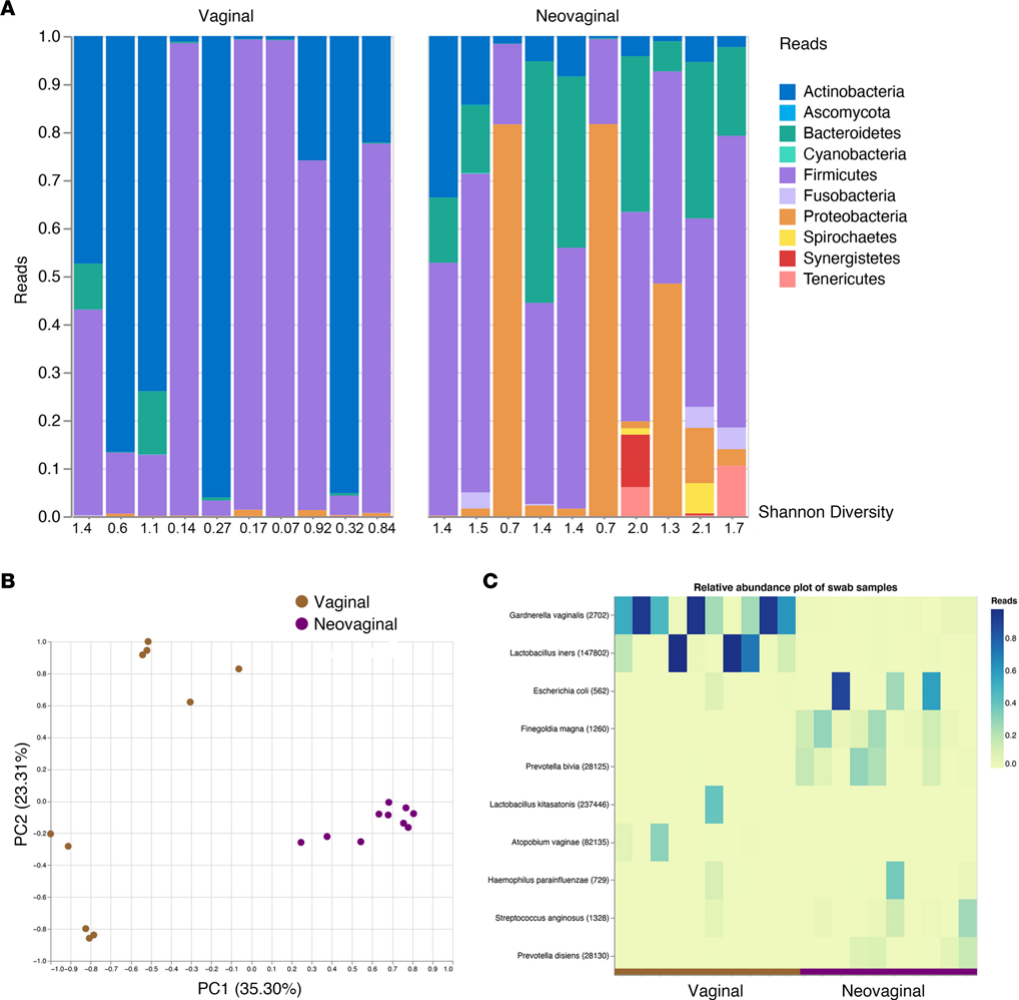
Microbial profiles determined by 16s rRNA sequencing revealed distinct microbial community structures between the vagina and neovagina. (**A**) Bar graph of phylum rank of vaginal microbiome in cisgender women (CW; *n* = 10) and neovaginal microbiome in transgender women (TGW; *n* = 10), including Shannon Alpha Diversity displayed below bar graphs, suggesting a different microbiome composition between the vaginal and the neovaginal compartment. (**B**) Neovaginal samples have a unique microbiome profile compared with vaginal samples based on principal component analyses. Brown circles, vaginal; purple circles, neovaginal. (**C**) Heatmap of relative abundance plot of species in neovaginal/vaginal compartment highlighting differences in compositions between vaginal and neovaginal microbiome.

**Figure 4:**
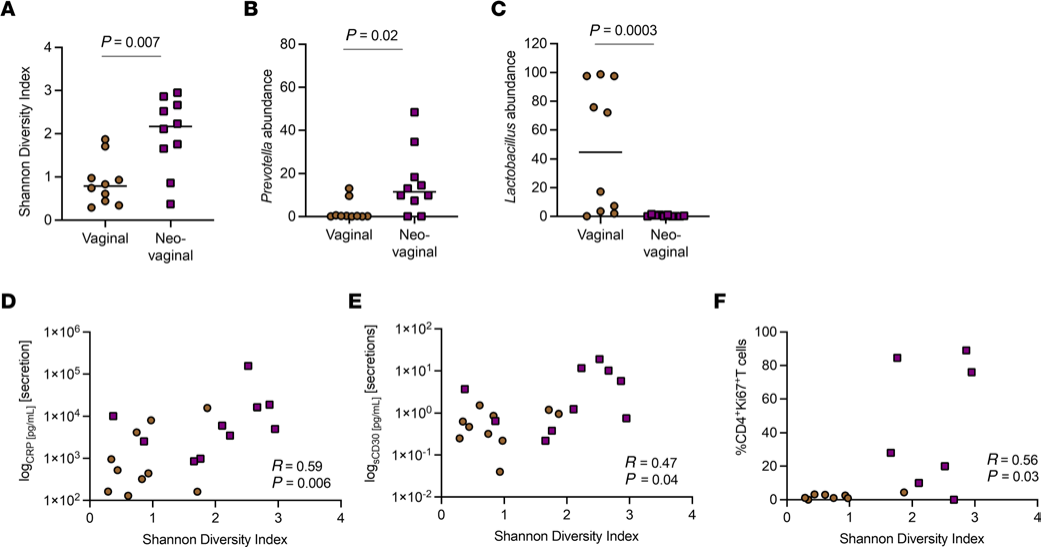
Distinct microbial community structures in the neovagina correlate with markers of local inflammation. Neovaginal (*n* = 10) microbial communities are characterized by a higher Shannon Diversity Index (**A**), a higher Prevotella abundance (**B**), and a lower *Lactobacillus* abundance (**C**) compared with vaginal (*n* = 10) microbial communities. The higher Shannon Diversity Index in the neovagina was linked to an increased inflammation profile, indicated by elevated neovaginal CRP and sCD30 levels (**D** and **E**). In addition, a direct correlation between the Shannon Diversity Index and the cycling of Ki67-expressing CD4^+^ T cells was observed (**F**). Differences between groups were analyzed using unpaired *t* tests. Spearman’s correlation was used to analyze associations between 2 variables. Brown circles, vaginal; purple squares, neovaginal.

Alterations of the mucosal environment in the FGT, such as inflammation and hormone levels, have been associated with an increased risk of HIV acquisition (24). The FGT microbiome has been closely associated with the inflammatory profile, with both the presence of specific taxa and their properties and activities being critical determinants of mucosal inflammation (32, 33). Based on those observations, we assessed the vaginal and the neovaginal microbiome utilizing 16s rRNA gene sequencing of swab samples. The composition of the neovaginal microbiome was significantly different from the vaginal microbiome on the phylum level, as indicated by a higher median Shannon Diversity Index of 1.4 compared with 0.6, respectively (*P* = 0.002; Figure 3A). These distinct differences were also supported by principal component analysis, indicating distinct clustering of all neovaginal samples compared with the vaginal microbiome samples (Figure 3B). The relative abundance of *Gardnerella vaginalis*, a notable component of the vaginal microflora, was observed in 80% of the vaginal samples compared with 0% of neovaginal samples (Figure 3C). Moreover, a species of lactobacilli, *Lactobaccilus iners*, was abundant in only vaginal but not neovaginal samples (Figure 3C). Distinct to the neovaginal compartment, we observed the abundance of *Finegoldia magna*, an anaerobic, Gram-positive bacterium that colonizes the skin and has been linked to inflammation (Figure 3C) (34, 35). We also observed a greater median Shannon Diversity Index on the species level in the neovaginal (2.2) compared with the vaginal microbiome (0.8, *P* = 0.0007, Figure 4A and Figure 3C). These findings support prior research showing that the microflora of the neovagina constitutes a mixed microflora of aerobe and anaerobe species usually found on the skin, in the intestinal microflora, or in a bacterial vaginosis microflora (35). We noted a high abundance of Prevotella in the neovaginal microbiome (*P* = 0.02; Figure 4B), which has been shown to be part of nonoptimal microbial communities in the vaginal microbiome of CW as well as in dysbiosis of the gut microbiome linked to inflammation in both instances (36). In contrast, the vaginal microbiome was largely dominated by *Lactobacillus* with a significantly higher abundance compared with the neovaginal microbiome (*P* = 0.0003; Figure 4C). Interestingly, the Shannon Diversity Index on the species level for the neovaginal/vaginal microbiome directly correlated significantly with the soluble markers of inflammation CRP (*r* = 0.59, *P* = 0.006; Figure 4D) and sCD30 (*r* = 0.47, *P* = 0.04; Figure 4E) as well as with the frequency of cycling (Ki67-expressing) CD4^+^ T cells (*r* = 0.56, *P* = 0.03; Figure 4F) in the respective compartments. This observation is in line with several studies that have shown an impact of the vaginal microbiome diversity on local inflammatory profiles (30, 37) and suggests a similar mechanism for the neovaginal microbiome, linking the higher bacterial diversity in the neovagina with higher local cellular and soluble inflammation.

### Increased frequency of CD4^+^CCR5^+^ T cells in the gut mucosa of TGW relates to changes in CCR5 DNA methylation.

**Figure 5:**
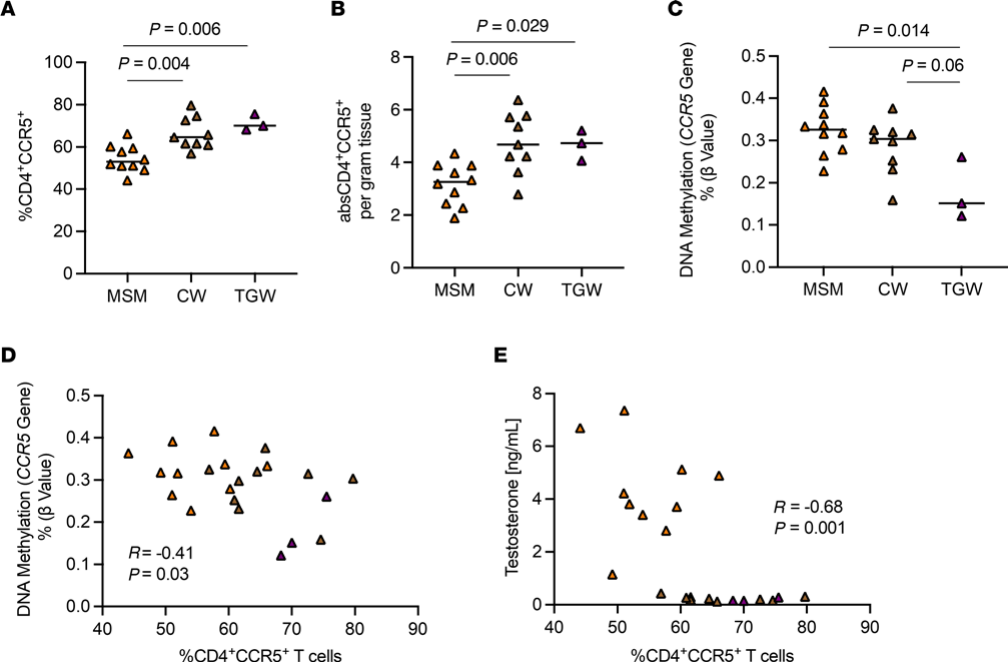
Frequency of CD4^+^CCR5^+^ T cells in the sigmoid colon in MSM (*n* = 10), CW (*n* = 9), and TGW (*n* = 3). The frequency (**A**) and absolute number (**B**) of colonic CD4^+^CCR5^+^ T cells was higher in CW and TGW compared with MSM. (**C**) Altered DNA methylation at loci cg22066626 at regulatory region of *CCR5* gene in the gut mucosa in TGW compared with CW and MSM. (**D**) DNA methylation levels related to *CCR5* gene were inversely associated with the frequency of gut mucosa CD4^+^CCR5^+^ T cells. (**E**) The frequency of colonic CD4^+^CCR5^+^ T cells was indirectly correlated with the plasma testosterone levels. Differences between groups were adjusted for multiple comparisons using Dunn’s test with Benjamini-Hochberg multiple comparison adjustment. Spearman correlation was used to analyze association between 2 variables. Orange triangles, MSM; brown triangles, CW; purple triangles, TGW.

TGW in this study and other studies (38) have reported engagement in neovaginal and/or in anal intercourse (Table 1 and Supplemental Figure 1B). Previous research has shown that the hormonal milieu and hormone levels can impact vaginal HIV susceptibility, with progesterone impacting the epithelial thickness and increasing the expression of the HIV co-receptor CCR5 on CD4^+^ T cells (7). However, little is known about hormonal effects on the gut mucosa and how potential changes might impact HIV susceptibility during anal intercourse. Therefore, as a first step, we explored the frequency of CD4^+^CCR5^+^ T cells in the gut mucosa of CW, TGW, and MSM. We observed that the frequency of CD4^+^CCR5^+^ T cells in the sigmoid colon was significantly higher in CW (median 64.5%) and in TGW (median 70.0%) compared with MSM (median 53.0%, *P* = 0.004 and *P* = 0.006, respectively; Figure 5A). This observation was verified when determining the absolute number of CD4^+^CCR5^+^ T cells per gram of tissue (median absolute numbers: MSM: 3.3 vs. CW: 4.7; *P* = 0.006 and MSM: 3.3 vs. TGW: 4.7; *P* = 0.029; Figure 5B). The epigenetic feature DNA methylation within cis-regulatory regions of the *CCR5* gene has been shown to correlate with CCR5 levels on T cells (39). Therefore, we examined DNA methylation states at the *CCR5* gene in the gut mucosa. We observed DNA at loci cg22066626 located within an annotated regulatory region of the *CCR5* gene was significantly hypomethylated in TGW compared with MSM and similar trends for CW (*P* = 0.014 and *P* = 0.06, respectively; Figure 5C). The DNA methylation levels related to the *CCR5* gene were inversely associated with the frequency of CD4^+^CCR5^+^ T cells in the gut mucosa (*r* = –0.41, *P* = 0.03), supporting the differences seen in CCR5 expression in the gut despite the low number of participants (Figure 5D). Interestingly, the frequency of CD4^+^CCR5^+^ T cells in the gut was inversely associated with the level of serum testosterone (*P* = –0.68, *r* = 0.001; Figure 5E), implying a potential impact of hormone levels on the expression of the HIV co-receptor CCR5 on CD4^+^ T cells. There was no correlation between estradiol E2 levels and the frequency of mucosal CD4^+^CCR5^+^ T cells observed (*P* = 0.22, *r* = 0.33; data not shown).

### Different rectal microbiome composition in TGW compared with MSM and CW suggests increased disruption of gut mucosal homeostasis and rectal inflammation.

**Figure 6:**
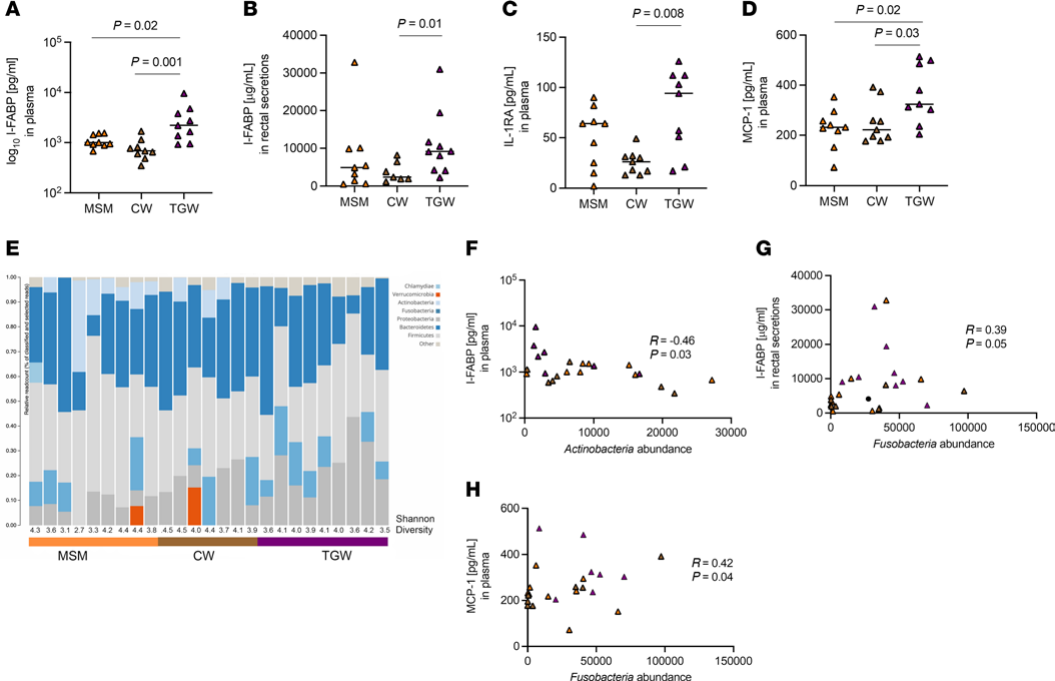
Differences in levels of soluble biomarkers in plasma/rectal secretions and rectal microbial profiles in MSM (plasma/rectal *n* = 9), CW (plasma *n* = 9, rectal *n* = 7), and TGW (plasma *n* = 9, rectal *n* = 10). The levels of I-FABP in (**A**) plasma and in rectal secretions (**B**) were increased in TGW compared with MSM and/or CW, indicating increased enterocyte damage. In addition, an increase in plasma IL-1RA (**C**) and MCP-1 (**D**) was observed in TGW compared with MSM and/or CW. (**E**) Bar graph of phylum rank of rectal microbiome in MSM (*n* = 9), CW (*n* = 7), and TGW (*n* = 9), including Shannon Diversity Index, suggesting differences in the level of Fusobacteria and Actinobacteria, with the abundance of Actinobacteria inversely correlating with plasma I-FABP levels (**F**) and the abundance of Fusobacteria directly correlating with rectal I-FABP (**G**) and plasma MCP-1 levels (**H**). Spearman correlation was used to analyze associations between 2 variables. Orange triangles, MSM; brown triangles, CW; purple triangles, TGW.

In addition to the assessment of mucosal cell populations that could contribute to the increased risk of HIV acquisition in TGW, we also determined 16 soluble biomarkers of inflammation, including markers indicative for microbial translocation (MTL) and enterocyte damage, such as Zonulin, sCD14, and intestinal fatty acid binding protein (I-FABP), in plasma and in rectal secretions. Plasma I-FABP levels were significantly increased in TGW (median: 2,199 pg/mL) compared with MSM (median: 884 pg/mL, *P* = 0.02) and CW (median: 681 pg/mL, *P* = 0.001). In rectal secretions I-FABP levels were also increased in TGW compared with CW (*P* = 0.01); however, there was no difference observed between TGW and MSM (Figure 6, A and B). Out of the inflammatory biomarkers tested, we found that plasma levels of IL-1RA and MCP-1 were increased in TGW compared with CW (*P* = 0.008 and *P* = 0.03, respectively) while only MCP-1 levels were increased in TGW compared with MSM (*P* = 0.02, Figure 6, C and D). No difference between MSM and CW was observed. Increased plasma levels of IL-1RA and MCP-1 have been previously linked to an increased risk of vaginal HIV acquisition (25, 40). There were no differences observed between plasma Zonulin and sCD14 between groups. Several studies have shown that the microbiome composition can impact local as well as systemic inflammation (41). We assessed the rectal microbiome by 16s rRNA in TGW (*n* = 9), MSM (*n* = 9), and CW (*n* = 7), and no differences at the phylum level were observed by Shannon Diversity Index, with a median index of 4.0, 3.8, and 4.1, respectively (data not shown). However, we observed less than 5% abundance of Actinobacteria in the rectal microbiome of all TGW compared with CW and MSM (Figure 6E). We also observed greater than 5% abundance of Fusobacteria in 7/9 TGW participants compared to 3/7 CW and 4/9 MSM. The abundance of Actinobacteria inversely correlated with plasma I-FABP levels (*r* = –0.46, *P* = 0.03; Figure 6F), while the abundance of Fusobacteria directly correlated with rectal I-FABP (*r*=0.39, *P* = 0.05; Figure 6G) and plasma MCP-1 (*r* = 0.44, *P* = 0.04; Figure 6H) levels, indicative of differing rectal microbiome composition in TGW compared with MSM and CW. This may disrupt mucosal homeostasis, as indicated by the increased enterocyte damage and an increased rectal inflammation evidenced by elevated MCP-1 levels.

## Discussion

TGW are among the most at-risk populations for HIV acquisition globally, which is still mainly attributed to increased sociodemographic and behavioral risk factors exacerbated by lack of gender-affirming care, decreased access to health care, and gender-based discrimination (42, 43). The main aim of the current study was to evaluate potential underlying biological risks of HIV acquisition in TGW, an important step toward further tailoring HIV prevention and treatment programs to this key population (44). To our knowledge, this study provides the first in-depth insight into a potential biological mechanism that could contribute to the higher risk of HIV acquisition in TGW based on the presence of CD4^+^CCR5^+^ target cells on the neovaginal surface reconstructed by penile skin inversion and the increased frequency of those target cells in the rectal mucosa.

We observed CD3^+^ T cells in the penile skin–lined neovagina, which is not too surprising, since human skin, as the largest and primary interface with the environment, contains large numbers of CD3^+^ T cells, with some of them residing in the epidermis (45, 46). In addition, several studies have shown that the inner and/or outer foreskin, potentially included in the reconstruction of the neovagina, is rich in CD3^+^ T cells (47, 48), thus posing an additional source of CD3^+^ T cells on neovaginal surfaces after GAS by penile skin inversion. However, the composition of CD3^+^ T cell subsets was vastly different on the neovaginal surface compared with the vaginal surface, showing a higher frequency of CD8^+^ and lower frequency of CD4^+^ T cells, with both subsets having increased metabolic activity and cell proliferation indicated by the expression of Ki67. Previous studies have shown that increased expression of Ki67 on cervical CD4^+^ T cells was linked to higher HIV susceptibility (27); thus, our results could indicate a greater risk of HIV acquisition in TGW engaging in neovaginal intercourse. The increased expression of PD-1 on CD8^+^ T cells could be indicative of further immune activation leading to immune exhaustion, which has been linked to an impaired response of mucosal PD-1^+^CD8^+^ T cells, e.g., to *Chlamydia trachomatis* (49). Additionally, neovaginal levels of the proinflammatory soluble activation markers CRP and sCD30 (50, 51) were increased compared with vaginal secretion levels, contributing to an overall proinflammatory neovaginal environment potentially further increasing HIV susceptibility. In CW the vaginal microbiome itself is an important determinant of the levels of local inflammation (25, 52, 53). Increased vaginal inflammation has been linked to bacterial vaginosis (BV) (54, 55), lowering the barrier for HIV infection, and this is in concordance with our observations in the neovaginal compartment showing a polymicrobial microbiome and an enrichment in Prevotella and Streptococcus, all associated with BV in CW (56). Those polymicrobial neovaginal communities were linked to increased inflammation in the neovaginal compartment. This observation is further corroborated by a recent study identifying neovaginas with diverse polymicrobial communities that elicit similar inflammation and host responses observed in BV in CW (24). In addition, we also observed the abundance of *Finegoldia magna* in the neovaginal compartment, which was previously described as part of the microflora of the penile skin-lined neovagina (35) and is otherwise known to colonize skin and nonsterile body surfaces. *Finegoldia magna* is known to activate neutrophiles through its soluble proteins FAF and L and induces a proinflammatory response (34), thus potentially contributing the a proinflammatory environment in the neovagina.

The abundance of CD4^+^CCR5^+^ target cells in the rectal mucosa serves as another major portal of entry for HIV (57, 58). Several studies demonstrated an increased risk of HIV acquisition and other STIs such as gonorrhea and chlamydia in MSM engaging in condomless anal intercourse (59, 60). Both nonhuman primate and human studies have linked this increased risk to the specific mucosal CD4^+^ T cells subsets highly susceptible to HIV infection, such as CD4^+^CCR5^+^ T cells and α_4_β_7_^hi^ memory CD4^+^ T cells and their activation status (61–63). In the current study, frequency and absolute cell count of CD4^+^CCR5^+^ T cells in the gut mucosa of TGW were significantly increased compared with MSM. Those findings were corroborated by the observation that the DNA methylation levels related to the *CCR5* gene in the gut were decreased in TGW, indicating increased gene expression (39, 64, 65). Furthermore, the increase in the frequency of mucosal CD4^+^CCR5^+^ T cells was directly linked to the lack of testosterone after GAS and is potentially also impacted by the usage of supraphysiologic doses of exogenous feminizing hormones (5). A similar association is known in the context of depo medroxyprogesterone hormonal contraception usage in enhancing risk of HIV acquisition in CW (66). Progesterone leads to thinning of the vaginal epithelium, and inhibiting secretion of cytokines and chemokines by innate and adaptive immune cells (11), while progestin-only contraceptives increase the expression of the CCR5 on CD4^+^ T cells in peripheral blood and vaginal mucosa (7, 9, 12). Given that TGW in this study reported engaging in both neovaginal and receptive anal intercourse, changes in both compartments are relevant to HIV transmission risk.

In addition to frequency of target cells, 2 critical determinants of the risk for HIV acquisition when engaging in anal intercourse are gut immune activation and the microbiota (67, 68). A normal gut microbiota is essential for immune homeostasis. Disruption in intestinal immunity can precipitate gut dysbiosis, which in turn leads to a disruption of the mucosal barrier, microbial translocation, and inflammation in the mucosa and periphery (69–72). Variations in the gut microbiota can be seen with changes in diet, antimicrobial use, stress, and environmental exposures (73). In addition, several studies have linked changes in the gut and stool microbiome in MSM to sexual preferences, with MSM engaging in receptive anal intercourse having a microbiome enriched for Prevotella species opposed to the microbiota of non-MSM enriched for Bacteroides (74, 75). We observed an association between higher abundance of Fusobacteria and lower abundance of Actinobacteria in TGW compared with MSM and CW with enterocyte damage, indicating a disruption of the mucosal homeostasis. Gut-derived Fusobacteria have been identified to have proinflammatory properties and are implicated in human colorectal cancer and chronic gut inflammation such as inflammatory bowel disease and have been associated with suboptimal immune recovery in HIV-infected patients (76–78). In contrast, Actinobacteria are major phyla of the gut microbiome, and though they represent only a small percentage, are pivotal in maintenance of the gut homeostasis (79).

One major limitation of our study is the modest sample size and the low number of TGW agreeing to undergo voluntary sigmoid biopsy. However, linking the actual frequency of mucosal CD4^+^CCR5^+^ T cells to mucosal DNA methylation status of the *CCR5* gene provides additional fidelity. Nevertheless, further studies with a more robust samples size would be required to further support these initial findings. Due to low acceptance of biopsy collection in this TGW population, cells from the vaginal/neovaginal compartment were collected using swabs instead of biopsy. It is known that 2 cervical swabs yield similar numbers of leucocytes compared to 1 biopsy; however, swabs and biopsies were biased toward macrophage and T lymphocytes, respectively (80), which should be considered when interpreting our results. Another caveat is the differing risk behavior of the CW enrolled in this study compared with TGW. While 40% of the TGW reported a history of sex work with an average of 35 sex partners, no CW reported history of sex work, with an average of 3 sex partners. BV leads to increased inflammation and is known to increase the risk of HIV acquisition (25, 53), with BV reaching a prevalence in female sex workers up to 70% (81). Therefore, we cannot exclude that CW with a similar risk behavior to the enrolled TGW would have been presented with a different vaginal microbiome composition, more resembling BV, which in turn can impact cellular and soluble markers of inflammation. We did not control for diet or other environmental factors in the microbiome analyses, and unmeasured confounding factors may explain the difference in the enrichment of Fusobacteria and Actinobacteria we are observing in TGW compared with CW and MSM. In addition, the effects of estrogen and progesterone have been studied extensively in the context of oral contraceptives in CW. Progesterone is known to induce thinning of the vaginal epithelium, inhibiting cytokine and chemokine secretion from T cells, macrophages, and dendritic cells and thus dampening the immune response (11). Conversely, estrogen might have a protective effect by decreasing CCR5 expression and inducing interferon-α and entry-mediated mechanisms (82, 83). For these reasons, we enrolled only CW not using hormonal contraceptives. However, systematic studies on the effect of exogenous hormones on the neovagina in TGW have not been reported, and our data are not able to account for potential biases such as different feminizing hormone treatments in our TGW cohort due to the small sample size. Finally, our findings are generally descriptive in nature, and further experiments or large studies will be necessary to determine the relevance of those mucosal findings in the neovagina and gut mucosa in TGW regarding their risk of HIV acquisition.

Overall, this study highlights the need to better understand potential biological factors unique to TGW that could influence the risk of HIV acquisition, such as increased inflammation due to GAS or changes pertaining to the continuous usage of supraphysiologic doses of exogenous feminizing hormones. The better understanding of those risks would be an important next step toward more comprehensive health care services tailored to the specific needs of transgender populations. In addition, future efficacy studies testing candidate preventive interventions should formally substratify these populations, as protective efficacy may vary among groups.

## Methods

### Study design and study procedures.

A cross-sectional study was conducted in 30 HIV-negative volunteers, including 10 TGW after GAS, by penile skin inversion, and taking exogenous hormones (estrogen with or without progesterone); 10 CW, not taking hormonal contraceptives; and 10 MSM. All volunteers underwent standardized clinical history questionnaires, physical exam, and phlebotomy. Volunteers had the option to consent to sigmoid biopsy, cervical/neovaginal, and/or rectal swab collection. The study was carried out within the RV304/SEARCH013 study cohort (ClinicalTrials.gov: NCT01397669) that is conducted at the Thai Red Cross AIDS Research Center and Institute of HIV Research and Innovation (IHRI) in Bangkok, Thailand. Participants were recruited at the Thai Red Cross Anonymous Clinic in Bangkok and Sisters Foundation in Pattaya. Eligible HIV-negative participants could undergo a onetime flexible sigmoidoscopy at King Chulalongkorn Memorial Hospital. Genital secretions were collected using swabs in participants who agreed to this optional procedure. The study was approved by the institutional review boards of Chulalongkorn University, Thailand, and the Walter Reed Army Institute of Research, US Army, United States. This study was conducted in a partnership with the Sisters Foundation in Pattaya (84) and included a series of focus groups to discuss this research regarding its relevance for the transgender community.

### Cell isolation from sigmoid biopsy and calculation of absolute numbers of colonic T cell subsets.

Participants underwent a routine sigmoidoscopy procedure under moderate conscious sedation. Approximately 30 endoscopic biopsies were randomly collected from the sigmoid colon using Radial Jaw 3 biopsy forceps (Boston Scientific), not accounting by visual control for potential collection of lymphoid aggregates, with 20–25 processed for flow cytometry analysis within 30 minutes of collection, as previously described (58). The cell count was done manually by trypan blue exclusion, which allows exclusion of epithelial cells due to their morphology compared with lymphocytes. Absolute numbers of CD4^+^ and CD4^+^CCR5^+^ T cells per gram of gut tissue were calculated by multiplying the total viable lymphocyte count by frequencies of cell subsets obtained from flow cytometric analysis. The total lymphocyte count per gram of tissue was calculated by dividing the viable lymphocyte count by the tissue weight. This proportion was then multiplied by the percentage of cells in the live lymphocyte gate, and that number was subsequently multiplied by the percentage of CD3^+^ lymphocytes. The absolute number of colonic CD3^+^ T cells was used in conjunction with the subset percentages to determine the absolute number of each T cell subset per gram of biopsy tissue.

### Cell isolation from vaginal/neovaginal swabs.

Two consecutive vaginal and neovaginal swabs were collected using FLOQSwabs (Copan Diagnostics) and placed into 15 mL sterile conical tubes containing 3 mL of RPMI media supplemented with 10% FBS media containing 1% HEPES, 1% l-glutamine, 1% penicillin/streptomycin, and 0.1% Amphotericin B (Life Technologies). Specimens were transported on ice and processed within 1 hour of collection by vigorously vortexing for 30 seconds to resuspend mucosal mononuclear cells (MMCs). The suspension was subsequently centrifuged at 630*g* at 4°C for 6 minutes and resuspended in 10% FBS media. Cell count was done manually by trypan blue exclusion, and cells were stained for flow cytometry analysis.

### Flow cytometry analysis.

Frequency and phenotype of peripheral blood and MMCs were determined as previously described (58). In brief, cells were stained with LIVE/DEAD Fixable Aqua Dead Cell dye (Thermo Fisher Scientific), blocked for Fc receptors using normal mouse serum (Thermo Fisher Scientific), and surface stained with antibody cocktail for 20 minutes. Samples were surface stained at room temperature for 30 minutes with the following antibodies: anti-CD3 PE-Cy7 (Invitrogen, catalog MHCD0312, clone 7D6, conc. 1:80), anti-CD4 ECD (Beckman Coulter, catalog 6604727, clone T4 conc. 1:80), anti-CD8 PerCP-Cy5.5 (BD Biosciences, catalog 341051, clone SK1, conc. 1:10), anti-CD38 APC (BD Biosciences, catalog 340439, clone HB7, conc. 1:80), anti–HLA-DR V450 (BD Horizon, catalog 642276, clone L243, conc. 1:80), and anti–PD-1 BV605 (BD Horizon, catalog 563245, clone EH12.1, conc. 1:40). For panels including anti-CCR5–APC-Cy7 (BD Pharmingen, catalog 557755, clone 2D7, conc. 1:80), surface staining was performed at 37°C. For samples including Ki67 antibodies, intracellular staining was performed by permeabilizing with 1× eBioscience Fixation/Permeabilization buffer (Thermo Fisher Scientific) for 60 minutes at 4°C before staining with anti-Ki67 PE (Invitrogen, catalog 12-5699-2, clone 20Raj1, conc. 1:160) for 30 minutes at 4°C. Samples were fixed in 1% paraformaldehyde before acquisition on a 4-laser custom-built LSR Fortessa (BD Biosciences) with at least 80,000 live cells acquired in the lymphocyte gate. Subsequent analysis was done using FlowJo software version 9.9.6 or higher.

### Soluble biomarker analysis.

Cytokine levels and biomarkers were measured in plasma and vaginal/neovaginal and rectal secretions as previously described (85). In brief, 18 cytokines and biomarkers were measured via a combination of customized Luminex-based multiplex assays (sCD30, sCD163, sgp130, sIL-6Ra, sTNF-R1, sTNF-R2, using Bio-Plex, Bio-Rad; and haptoglobin, CRP, IFN-α2, IL-1RA, IL-8, IL-10, MCP-1, MIP-1β, RANTES, using Milliplex MAP, MilliporeSigma) and single-plex ELISAs (I-FABP, sCD14, using Human Quantikine ELISAs, R&D Systems) per manufacturers’ protocols. To account for variances in samples collected from mucosal swabs, total IgA was quantified by ELISA (Millipore Sigma), and levels of soluble markers were normalized to total IgA.

### DNA extraction and PCR amplification.

Vaginal, neovaginal, and rectal swabs were collected using FLOQSwabs (Copan Diagnostics) and stored at –80°C. For assessment of the microbiome ZymoBIOMICS DNA Microprep Kit was utilized for a standardized cellular lysis of swabs and to isolate bacterial DNA for 16s library preparation. The kit utilizes a bead-beat sample, spin-column purification, and filter to remove PCR inhibitors. DNA was quantified utilizing a NanoDrop instrument (Thermo Fisher Scientific), and 50 ng total DNA was input into 16s library construction.

### Next-generation sequencing and data processing.

For vaginal and neovaginal swabs, the Swift Biosciences 16s+ ITS panel was utilized to generate sequencing libraries. A total of 25 nanograms of DNA was utilized for input into a multiplex PCR targeting all variable regions of the 16s rRNA gene. Dual-indexed Illumina sequencing adapters were utilized to barcode samples. Quantitative PCR was utilized to quantify libraries for pooling. Sequencing was performed on an Illumina iSeq 100 system generating 2 × 150 bp paired-end reads. For rectal swabs, a 2-step PCR protocol targeting the V4 region of the 16s rRNA gene was utilized to generate 16s libraries according to published protocols (86). Sequencing was obtained on an Illumina MiSeq system generating 2 × 300 bp paired-end reads. The raw sequencing data generated were processed by filtering raw fastq files for primer an adapter dimer sequences, then removing contaminating host sequences, and analyzed using both Qiime 1.9.1 analysis software and the One Codex data platform. Sequencing data were uploaded to the NIH NCBI GEO database (accession number GSE234969).

### Epigenetic DNA methylation profiling of gut tissues.

For Illumina EPIC Array-Based DNA Methylation Analysis, 500 ng of DNA per sample were isolated from biopsies lysed in RLT buffer using the QIAGEN TissueRuptor II and AllPrep DNA/RNA/miRNA Universal Kit for all types of tissues. DNA was bisulfite-converted using the EZ DNA Methylation kit (Zymo Research) according to the manufacturer’s instructions. Bisulfite-converted DNA samples were randomly assigned to a chip well on the Infinium HumanMethylationEPIC BeadChip, amplified, hybridized onto the array, stained, washed, and imaged with the Illumina iScan SQ instrument to obtain raw image intensities. Raw Methylation EPIC array IDAT intensity data were loaded and preprocessed in the R statistical programming language (http://www.r-project.org) using the Chip Analysis Methylation Pipeline (ChAMP, version 2.8.3). IDAT files were loaded using the champ.load function. All samples passed quality control metrics. Comprehensive filtering was applied to the data set for probes with detection *P* values < 0.01, all non-CpG probes, previously published SNP-related probes, multihit probes, and probes on sex chromosomes. Methylation β-values ranging 0–1 (corresponding to unmethylated to methylated signal intensity) for each sample were normalized using the BMIQ function implemented in the ChAMP pipeline. DNA methylation loci related to the *CCR5* gene were annotated using the EPIC array R package annotation IlluminaHumanMethylationEPICanno.ilm10b4.hg19. Methylation β-values were utilized and represent a quantification at each CpG site of β = *M*/(*M* + *U* + *a*), where *M* > 0 and *U* > 0 denote the methylated and unmethylated signal intensities.

### Statistics.

All statistical analysis was performed using Prism version 8.2.0 for Mac OS (GraphPad Software). The paired/unpaired *t* test was used for between group comparisons, while Kruskal-Wallis was used for comparison between more than 2 groups. Spearman’s rank test was used to evaluate associations. All values reported were median (interquartile range). Statistical tests were 2 sided with *P* ≤ 0.05 considered statistically significant. For multiple comparisons Dunn’s test was performed with Benjamini-Hochberg multiple comparisons adjustment.

### Study approval.

All studies were approved by the Chulalongkorn University and Walter Reed Army Institute of Research institutional review boards for human research. Written informed consent was obtained from all participants prior to inclusion in the studies. The investigators have adhered to the policies for protection of human participants as prescribed in AR-70-24 (Use of volunteers as subjects of research, US Army Regulation: Research and Development).

### Data availability.

Data are available from the corresponding author upon request or in the provided Supporting Data Values file. Sequencing data were uploaded to the NIH NCBI GEO database (accession number GSE234969).

## Author contributions

Study design and study procedures were developed by AS, CS, S Sriplienchan, EK, NP, TN, TW, LCN, and SV. Cohort oversight was the responsibility of CS, S Sriplienchan, NR, and NP. Mucosal secretion collection/oversight was the responsibility of CS, S Sriplienchan, and N Teeratakulpisan. Sigmoid biopsy collection oversight was the responsibility of RR. Cell isolation from sigmoid biopsies, vaginal/neovaginal swabs, and flow cytometry were the responsibility of YPN, N Tragonlugsana, and S Sukhumvittaya. Next-generation sequencing and epigenetic DNA methylation were the responsibility of PE, RT, APSP, and MJC. Soluble biomarker analysis was the responsibility of BMS and SK. Data analysis/interpretation was the responsibility of AS, MJC, S Sukhumvittaya, SP, SV, BMS, SA, SK, and LCN. The original draft was written by AS, SV, and MJC. All authors have reviewed, edited, and approved the manuscript.

## Supplementary Material

Supplemental data

Supporting data values

## Figures and Tables

**Table 1:**
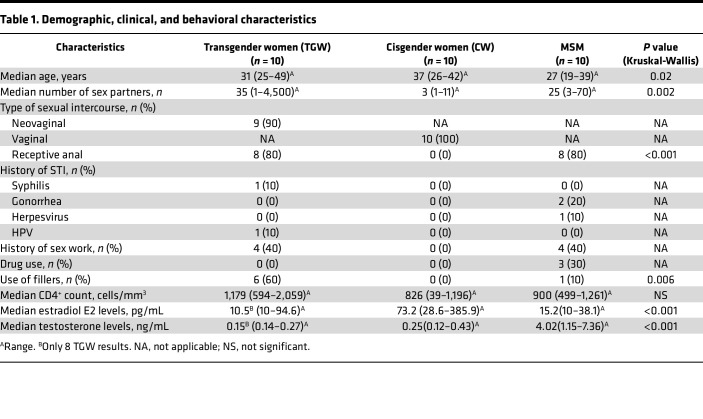
Demographic, clinical, and behavioral characteristics

## Version 1 07/11/2023

In-Press Preview

## Version 2 08/22/2023

Electronic publication
