# Supplementary material for: Distinct mucosal and systemic immunological characteristics in transgender women potentially relating to HIV acquisition
Source: JCI Insight. 2023 Aug 22;8(16):e169272. doi: 10.1172/jci.insight.169272 (PMC10543719; doi:10.1172/jci.insight.169272)
Supplement: Supplemental data [file jciinsight-8-169272-s118.pdf]

Supplemental materials

Supplemental Figures

A

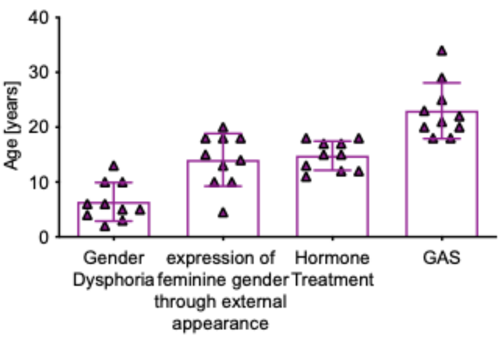

B

Intercourse [%]	CW	MSM	TGW
receptive vaginal only	100	0	20
receptive anal only	0	100	10
both	0	0	70

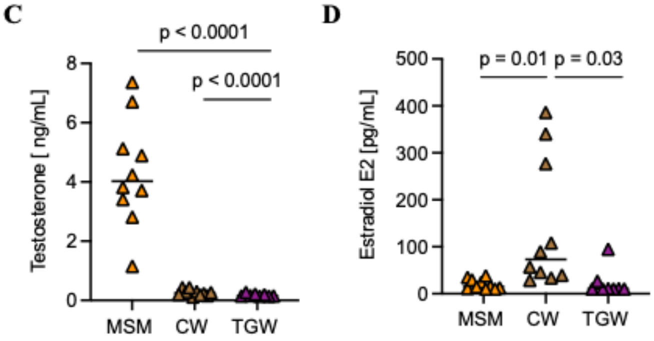

Figure S1. *Study participant characteristics.* (**A**) Age of gender dysphoria, expression of feminine gender through appearance, initiation of feminizing hormone treatment and gender affirmation surgery (GAS) in 10 enrolled transgender women (TGW, n=10). (**B**) Frequency of reported intercourse in TGW, cisgender women (CW, n=10) and men who have sex with men (MSM, n=10). (**C**) Level of serum Testosterone, and (**D**) serum Estradiol E2 in MSM, CW and TGW. Difference between groups were analyzed unpaired t test (C+D). Orange triangles - MSM, brown triangles – CW, purple triangles – TGW

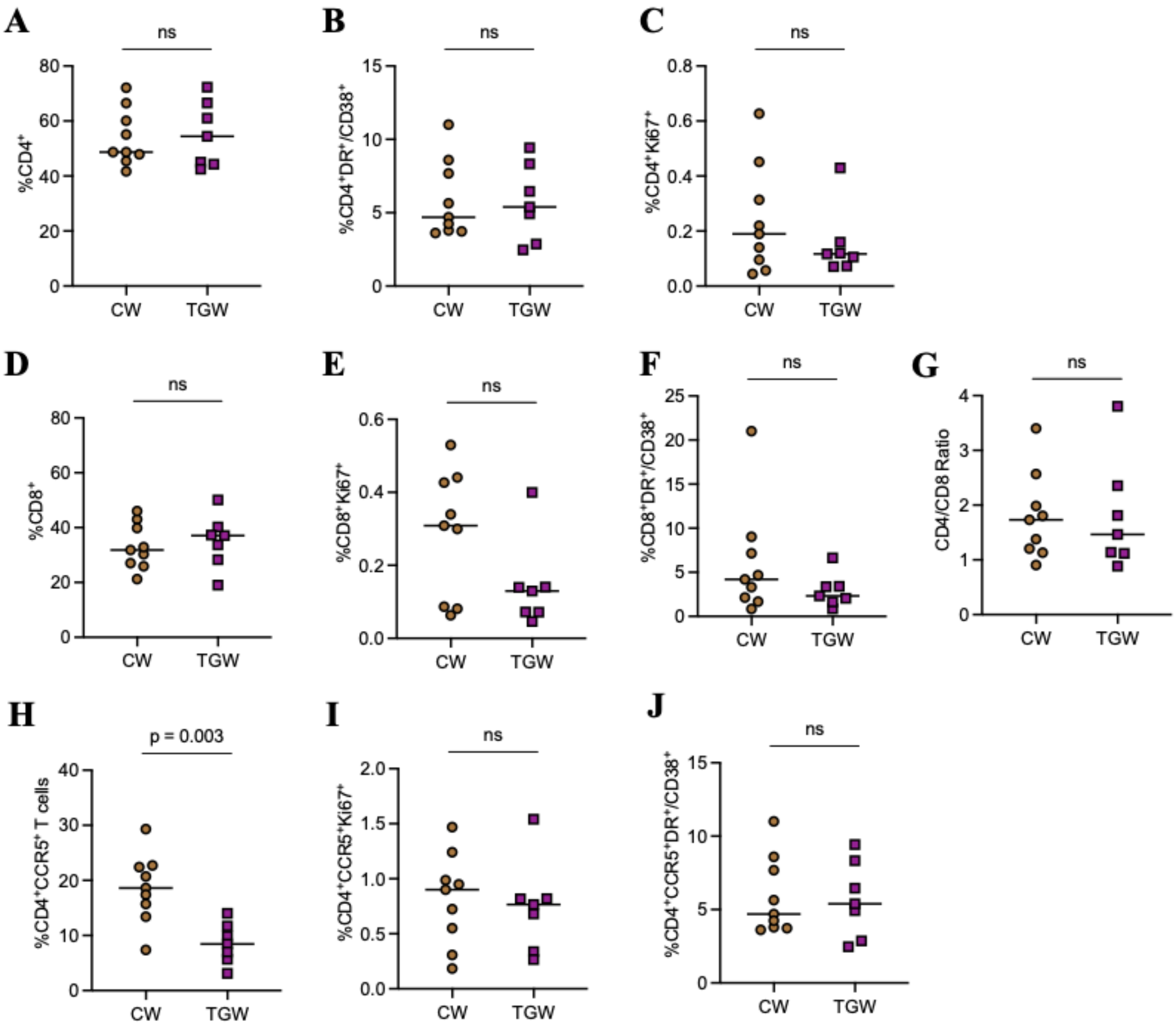

Figure S2. *Frequency, activation (HLA-DR^{+}/CD38^{+}) and cycling (Ki67^{+}) status of peripheral blood CD4^{+}, CD4^{+}CCR5^{+} and CD8^{+} T cells between CW (n=9) and TGW (n=7).* There were no differences in the frequencies of peripheral CD4^{+} T cells (**A**), their activation (**B**) or cycling status (**C**) observed between cisgender (CW) and transgender women (TGW). The same was observed for the frequency (**D**), activation (**E**) and cycling status (**F**) of CD8^{+} T cells. (**G**) No difference in peripheral CD4/CD8 T cells ratio between CM and TGW. There was a higher frequency of peripheral CD4^{+}CCR5^{+} T cells observed in TGW (**H**), however there was no difference in the activation (**I**) or cycling (**J**) status of CD4^{+}CCR5^{+} T cells. ns: not statistically significant. Difference between groups were analyzed using unpaired t test.

Supplemental Acknowledgments
RV304/SEARCH013 Study Team
Pathariya Promsena, SEARCH Research Foundation, Bangkok, Thailand
Nitiya Chomchey, SEARCH Research Foundation, Bangkok, Thailand
Duanghathai Suttichom, SEARCH Research Foundation, Bangkok, Thailand
Jintana Intasan, SEARCH Research Foundation, Bangkok, Thailand
Ponpen Tantivitayakul, SEARCH Research Foundation, Bangkok, Thailand
Ratchapong Kanaprach, SEARCH Research Foundation, Bangkok, Thailand
Eugene Kroon, SEARCH Research Foundation, Bangkok, Thailand
Suthat Chottanapund, SEARCH Research Foundation, Bangkok, Thailand
Praphan Phanuphak, Institute of HIV Research and Innovation, Bangkok, Thailand
Rapee Trichavaroj, Institute of HIV Research and Innovation, Bangkok, Thailand
Kiat Ruxrungtham, Department of Medicine, Faculty of Medicine, Chulalongkorn University, Bangkok, Thailand
Sunee Sirivichayakul, Department of Medicine, Faculty of Medicine, Chulalongkorn University, Bangkok, Thailand
Netsiri Dumrongpisutikul, Department of Medicine, Faculty of Medicine, Chulalongkorn University, Bangkok, Thailand
Nelson Michael, Center for Infectious Diseases Research, Walter Reed Army Institute of Medical Research, Silver Spring, Maryland, USA
Sheila Peel, Center for Infectious Diseases Research, Walter Reed Army Institute of Medical
Julie Ake, US Military HIV Research Program, Walter Reed Army Institute of Research, Silver Spring, Maryland, USA.
Merlin Robb, US Military HIV Research Program, Walter Reed Army Institute of Research, Silver Spring, Maryland, USA.
Jessica Cowden, Armed Forces Research Institute of Medical Sciences, Bangkok, Thailand
Siriwat Akapirat, Armed Forces Research Institute of Medical Sciences, Bangkok, Thailand
Bessara Nantapinit, Armed Forces Research Institute of Medical Sciences, Bangkok, Thailand
